# Utility of exome sequencing for the diagnosis of pediatric-onset neuromuscular diseases beyond diagnostic yield: a narrative review

**DOI:** 10.1007/s10072-023-07210-z

**Published:** 2023-11-22

**Authors:** Martha Cecilia Piñeros-Fernández, Beatriz Morte, José Luis García-Giménez

**Affiliations:** 1 Servicio de Neurología Pediátrica, Hospital Pediátrico, Fundación Cardio Infantil-LaCardio, Bogotá, Colombia; 2 Unidad Pediátrica, Los Cobos Medical Center, Bogotá, Colombia; 3 Consulta Externa Especializada, Virrey Solís IPS, Bogotá, Colombia; 4 Centro de Investigación Biomédica en Red de Enfermedades Raras (CIBERER), Instituto de Salud Carlos III, Madrid, Spain; 5 Instituto de Investigación Sanitaria INCLIVA, Valencia, Spain; 6 Departamento de Fisiología, Facultad de Medicina y Odontología, Universitat de València, València, Spain

**Keywords:** Diagnostic yield, Neuromuscular disease, Next-generation sequencing, Exome, Pediatric patients

## Abstract

Diagnosis of neuromuscular diseases (NMD) can be challenging because of the heterogeneity of this group of diseases. This review aimed to describe the diagnostic yield of whole exome sequencing (WES) for pediatric-onset neuromuscular disease diagnosis, as well as other benefits of this approach in patient management since WES can contribute to appropriate treatment selection in NMD patients. WES increases the possibility of reaching a conclusive genetic diagnosis when other technologies have failed and even exploring new genes not previously associated with a specific NMD. Moreover, this strategy can be useful when a dual diagnosis is suspected in complex congenital anomalies and undiagnosed cases.

## Introduction

Genetic NMDs are a heterogeneous group of diseases caused by pathogenic variants in coding genes for structural proteins of muscles, neuromuscular junction, lower motoneuron, or peripheral nerves (Table 1) [1]. Most NMDs are considered rare diseases [2]. A study carried out in the North of England reported the prevalence of pediatric-onset NMD at 36.9 per 100,000 [3]. There are reports of studies of specific prevalence for different groups of NMD with a pediatric onset: The group of motor neuron diseases such as amyotrophic lateral sclerosis (ALS) is extremely rare in pediatric age with a prevalence of 0.008 cases per 100,000 people [4], while the prevalence for 5q-related spinal muscular atrophy (SMA) is 1 to 2 per 100,000 people for all types of SMA [5]. The overall prevalence of congenital myopathy varies from 1.6 to 2.8 per 100,000 [6], and the prevalence of Duchenne muscular dystrophy (DMD) ranges between 1.7 and 5.3 per 100,000 people based on different reports [7, 8].

**Table 1:** Classification of neuromuscular diseases

Nº NMD group	Diseases	Nº associated genes
1	Muscular dystrophies	57
2	Congenital muscular dystrophies	36
3	Congenital myopathies	47
4	Distal myopathies	22
5	Other myopathies	37
6	Myotonic syndromes	6
7	Ion channel diseases	8
8	Malignant hyperthermia	2
9	Metabolic myopathies	30
10	Hereditary cardiomyopathy	112
11	Congenital myasthenic syndromes	35
12	Motoneuron diseases	91
13	Hereditary ataxias	85
14	Hereditary peripheral sensitive and motor neuropathies	109
15	Hereditary spastic paraplegia	67
16	Other neuromuscular disorders	75

Source: Benarroch et al. [1]

A computerized version of the table is freely accessible at http://www.musclegenetable.fr. Currently, the table contains 1173 diseases and 658 different genes

A particular challenge in the diagnostic journey of patients with suspected NMD is genetic heterogeneity, which encompasses not only disease phenotypes but also the age of disease onset and the broadly overlapping clinical features faced by clinicians. Beyond this, there is also broad genetic variability, in which a pathogenic variant of the same gene is related to different clinical groups of NMD, and in the same disease, a clinical phenotype could have autosomal dominant, autosomal recessive, X-linked, and non-mendelian (e.g., mitochondrial) modes of inheritance [1].

The diagnostic yield of genetic tests is defined as the percentage of patients in whom a particular phenotype with a pathogenic variant is found in the gene, exome, or genome analyzed [9]. In general, the diagnostic yield of a test depends on disease prevalence and can vary temporally and geographically according to population and clinical setting [29]. Focusing on NMD, reports of exome diagnostic yield in NMD in the general population are variable, and depending on the group or groups of NMD analyzed, this yield fluctuates between 12.9% in a heterogeneous group of NMD patients in the Haskell et al. study [10] to 45% obtained by Ghaoui et al. [11] in a study focused specifically on patients with limb-girdle muscular dystrophy from a population with a high percentage of consanguinity. Ghaoui et al. clarify that the diagnostic yield was 60% in trio exome tests versus 40% in singleton tests [11]. In comparison, genetic panels for NMD yield between 30 and 49% in non-related populations [12–15] and 63% in populations with high consanguinity [16].

Whole-genome sequencing yields 43% in pediatric patients with neurological diseases, a percentage which increases to 62.5% in neuromuscular disease patients [17]. Transcriptomic studies of muscle tissues yield 36–38%, detecting causal genes from variant calling and aberrant splicing analysis [18]. Exome sequencing data reanalysis has been reported to increase the diagnostic yield of the exome in undiagnosed cases [19]. In the diagnosis of neurologic rare diseases, it is possible to increase diagnostic yield by up to 20% by applying a systematic and collaborative genomic data reanalysis approach that includes high-quality phenotyping, data reprocessing, results reinterpretation, and data sharing [20]. After genotype-guided diagnostic reassessment and complementary investigations of NMD patients who underwent exome sequencing, the yield increases by 7.5% [21].

## Methods

We performed a narrative review, defining diagnostic yield as the proportion of positive results obtained from the study population through exome sequencing diagnostic testing.

Eligible study types included cohort, case–control, follow-up, prospective and retrospective clinical trials, and transversal studies in which patients with pediatric-onset neuromuscular diseases were diagnosed through whole exome sequencing or clinical exome.

The databases used were PubMed, EBSCO, and Web of Science. Lexical items and syntax were adjusted in each database consulted. The following search terms were used.

PubMed: (child*) OR (pediatric)) AND (neuromuscular disease) OR (neuromuscular disorder) AND (exome) OR (whole exome) OR (whole exome sequencing) AND (diagnostic yield).

Ebsco: TI pediatric AND TI neuromuscular disease OR TI neuromuscular disorders AND TI exome OR TI exome sequencing AND TI diagnostic yield.

Web of Science (WoS): TS = (child OR pediatric OR pediatric) AND TS = (neuromuscular disease OR neuromuscular disorder) AND TS = (whole exome OR exome OR whole exome sequencing AND diagnostic yield).

## Results

**Fig. 1:**
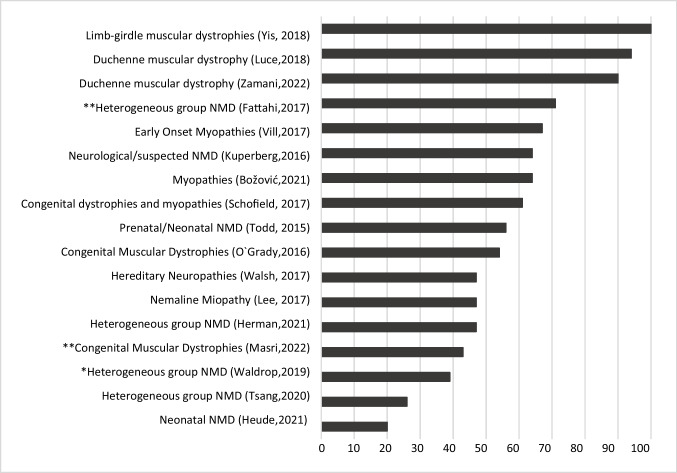
Diagnostic yield of WES in NMD reported by studies included in the narrative review. Studies of a heterogeneous group of NMDs reported a diagnostic yield below 50%. Although the studies are different from each other, note that diagnostic yield has not necessarily improved in more recent studies. *WES trio. **Consanguinity

Titles and abstracts were screened for potential relevance, selecting those written in Spanish or English, which included a specified diagnosis of neuromuscular disease through exome sequencing and which met the inclusion criteria. The search results in the three databases returned a total of 1112 citations: (Pubmed 1108 citations, EBSCO 315 citations, and WoS 114 citations). After removing duplicates and reading the titles and abstracts, 36 articles were selected for complete text reading, with 17 studies remaining eligible for review. The characteristics of the 17 studies finally included in this narrative review are summarized in Table 2, while Fig. 1 shows the diagnostic yield for the studies included, from highest to lowest performance.

**Table 2:** Summary of studies included in the narrative review

Nº	Author	Country	Year	Type of disease	*N* exome	*N* positive exome	*Dx* yield %	Ref
*Diagnostic yield of exome in populations with clinically different NMD*								
1	Tsang	China	2020	Heterogeneous group of NMD	50	13	26	[22]
2	Herman	USA	2021	Heterogeneous group of NMD	79	37	47	[23]
3	Waldrop	USA	2019	Heterogeneous group of NMD^*^	31	12	39	[24]
4	Fattahi	Iran	2017	Heterogeneous group of NMD Consanguinity 58%	37^a^	27	73	[25]
5	Kuperberg	Israel	2016	Heterogeneous group of non-diagnosed neurological diseases	11	7	64	[26]
*Diagnostic yield of exome in congenital muscular dystrophy and congenital myopathy*								
6	Masri	Jordan	2022	Congenital muscular dystrophy Consanguinity 73%	44	19	43	[27]
7	Schofield	Australia	2017	Congenital dystrophy and congenital muscular dystrophy	26	16	61	[28]
8	Vill	Germany	2017	Early-onset myopathies	12	8	67	[29]
9	O’Grady	Australia	2016	Congenital muscular dystrophy	22	12	54	[30]
10	Lee	Korea	2017	Nemaline myopathy	15	7	47	[31]
*Diagnostic yield of exome in muscular dystrophy*								
11	Yis	Turkey	2018	Limb-girdle muscle dystrophy	7	7	100	[32]
12	Zamani	Iran	2022	Duchenne muscular dystrophy	40	36	90	[33]
13	Luce	Argentine	2018	Duchenne muscular dystrophy	38	36	94	[34]
14	Božović	Slovenia	2021	Muscular diseases	22	14	64	[35]
*Diagnostic yield of exome in hereditary neuropathies*								
15	Walsh	Australia	2017	Hereditary neuropathies	15	7	47	[36]
*Diagnostic yield of exome in prenatal and neonatal age*								
16	Todd	Australia	2015	NMD detected at prenatal and neonatal age	23	13	56	[37]
17	Heude	France	2021	Patients with neonatal respiratory distress	5	1	20	[38]

*Dx yield*: diagnostic yield; *N*, number of cases studied; *WES*, whole exome sequencing

*Trio exome

### Diagnostic yield of exome in populations with clinically different NMD

Multiple relevant studies describing WES for different NMDs have shown wide variation in exome diagnostic yield. In a study of WES performed on 50 pediatric patients with NMD, Tsang et al*.* reported a diagnostic yield of 26%, which varied depending on the NMD group. For example, for congenital myopathy, the diagnostic exome yield reached 17% (four of 24 patients, with causal variants in the *ACTA1* gene in two patients, the *SELENON* gene in one patient, and the *DNM2* gene in one patient); for congenital muscular dystrophy, the diagnostic exome yield reached 36% (4/11 patients with pathogenic variants in the *COL6A1* gene in two patients, *LMNA* in one, *POMT1* genes in one*,* and *LAMA2* in one); for hereditary peripheral neuropathy, the diagnostic yield was 27% (3/11 patients with pathogenic variants in the *SCN11A* gene in one patient and the *GJB1* gene in two); and the diagnostic yield for complex condition NMD reached 25% (1/4), identifying a genetic variant in the *TGB1* gene associated with Camurati-Engelman syndrome. Tsang et al*.* also compared the diagnostic yield of exome analysis and genetic panels, obtaining comparable results (26% and 24%, respectively) [22].

In contrast, Herman et al*.* reported a diagnostic yield of 47% in 79 of 106 pediatric patients who underwent clinical exome sequencing. They confirmed the diagnosis of patients with juvenile amyotrophic lateral sclerosis (*FUS*), Bethlem myopathy (*COL6A1*), central core disease (*RYR1*), congenital myasthenic syndrome type 4C (*CHRNE*), congenital myasthenic syndrome type 10 (*DOK7*), Emery-Dreifuss muscular dystrophy (*LMNA*), Harel-Yoon syndrome (*ATAD3A*), limb-girdle muscular dystrophy (*FKRP, POMT1, TNPO3, TTN*), mitochondrial depletion syndrome (*MTFMT, TK2*), myofibrillar myopathy (*BAG3*), nemaline myopathy (*ACTA1*), Stuve-Wiedemann syndrome (*LIFR*), and 10 novel pathogenic variants in nine known disease-associated genes: *AIFM1, CHRND, COL12A1, GARS, MAGEL2, MECP2, LIFR, PRDM16*, and *TTN*. In two cases, patients had complex diseases: One patient had pathogenic variants in *MAGEL2* and *CHRND*, which are genes associated with Schaaf-Yang syndrome and congenital myasthenic syndrome, respectively. Another patient showed composed heterozygosity for both *RYR1* and *TTN*, which supports a diagnosis of central core disease and LGMD, respectively [23].

Waldrop et al*.* conducted their study using a strategy based on trio exome sequencing for NMD patient diagnosis, obtaining a genetic diagnosis in 39% of patients (12/31). The pathogenic variants confirmed in the study were the *ACTA1* gene in one patient, the *EPG5* gene in three, the *TBCK, TTN, DYNC1H1, STAC3, IGHMBP2,* and *KLHL40* genes in one patient each, and two genes causing non-neuromuscular diseases, the *EXOSC8* in one patient with pontocerebellar hypoplasia type 1C and the *EIF2B5* in one patient with vanishing white matter disease [24].

Fattahi et al*.* reported consanguinity of 52% in progenitors of the 45 NMD patients included in their study; 37 patients reported with pediatric-onset NMD. The diagnostic yield of exome sequencing for pediatric-onset NMD was 73%. Importantly, 50% of diagnosed patients had a phenotype disease of LGMD. Pathogenic variants found in patients with pediatric-onset NMD were associated with the following genes: the *DMD* gene in six patients, the *SGCA* and *SGCB* gene in two patients each, the *ISPD* gene in one, the *LAMA2* gene in two, the *CAPN3* gene in seven patients, the *COL6A1*, *COL6A3*, *LMNA*, *PLEC1*, *SYNE1*, *TNNT2,* and *GJB1* in one patient each, and the *DMD* gene in three patients [25].

Kuperberg et al*.* analyzed 28 pediatric patients with undiagnosed neurological diseases, including 11 patients with suspected NMD. The diagnostic yield in the subgroup with suspected NMD was the highest in the study, at 64%. The authors associated their results with the small number of patients studied for NMD. The study also showed interesting findings obtained in two patients with weakness but non-neuromuscular diseases, one patient diagnosed with Schwachman-Diamond syndrome, and the second patient with a mutation in the *TGBF1* gene which causes Camerutti-Engelman syndrome, an autosomal disorder characterized by bone dysplasia and pain. Note that another patient was diagnosed with congenital myasthenia caused by a mutation of the *CHRNE* gene, so the empirical treatment for a previous mitochondrial disease diagnosis was stopped and replaced by a new treatment for myasthenia [26].

### Diagnostic yield of exome in congenital muscular dystrophies and congenital myopathies

Masri et al*.* performed WES as the first-tier genetic diagnostic strategy, carrying out a prospective study of singleton WES in 44 children with suspected CMD, identifying consanguinity in 76.7% of patients. The authors found pathogenic/likely pathogenic variants related to congenital muscular dystrophies in 43.1% (19/44) of patients. Interestingly, 5/44 patients had pathogenic or likely pathogenic variants in NMD genes other than CMD: one variant in the *HSPG2* gene related to Shwartz-Jampell syndrome, one variant in the *GDAP* gene related to hereditary sensory motor neuropathy, and one in *SIGMAR1* gene related to distal spinal muscular atrophy. In addition, they clarified that two patients had non-neuromuscular diseases, related to *NSUN2* and *POLR3A* genes, respectively [27].

Interestingly, Schofield et al. [28], Vill et al*.* [29], and O’Grady et al. [30] reported comparative results from different methods used for genetic diagnosis of congenital muscular dystrophy, congenital myopathy, or both (Table 3). Schofield et al. reported a diagnostic yield of 61% performing WES in 26 of 56 patients. Causative variants were found in nine patients with congenital muscular dystrophy in the *ACTA1* gene (in one patient), *GFPT1* (in one patient), *PIGY* (in two patients), *POMT1* (in one patient), *MCU1* (one patient), *RYR1* (one patient), and *TTN* (one patient). In seven patients with nemaline myopathy, the authors identified several causal variants in the *ACTA1* gene (in one patient), the *LMOD3* gene (in two patients), the *PLOD1* gene (in one patient), and the *NEB* gene (in four patients) [28].

**Table 3:** Comparison of diagnostic yield of genetic tests in CMD and CM

Author	*n*			Diagnostic yield %		
	Traditional method	Exome	Genetic panels	Traditional method	Exome	Genetic panels
Schofield^a^ [28]	26(56)	44(56)	42(56)	46	79	75
O’Grady^b^ [30]	41(122)	12(22)	10(11)	33.6	54	91
Vill^c^ [29]	31(56)	8(12)	23(44)	55	67	52

The diagnostic yield of exome and genetic panels is higher than the traditional method for congenital muscular dystrophies and congenital myopathies. Diagnostic yield in Schofield’s study increased to 79%, assuming that patients diagnosed with CMA would also be diagnosed by genetic panels or WES. O’Grady’s study extends over a long period with patients widely evaluated, and genetic panels were done in 11 cases

^a^Congenital muscular dystrophies and congenital myopathies (CMD) study

^b^Congenital myopathies (CM) study

^c^Early onset myopathies’ study

Vill et al*.* retrospectively analyzed results from 98 probands with early-onset myopathies, combining deep clinical phenotyping, muscle imaging, and molecular genetic analysis. An NGS myopathy-related genetic panel was performed in 44 patients, which allowed the diagnosis in 52% of cases (23/44). In contrast, by using WES, it was possible to reach a diagnosis in about eight cases out of 12, reaching a diagnostic yield of 67% [29] (Table 3). About 25% of patients had congenital myopathies, and 44% of patients had congenital muscular dystrophy. The authors were able to confirm pathogenic variants through the WES testing approach in the *MTM1* gene (in one patient), *RYR1* gene (in three patients), the *Col6A2* gene* (*in one patient), *NEB* (in one patient), *ACTA1* (in one patient), and *POMT1 (*in one patient) [29].

Likewise, O’Grady et al. analyzed a cohort of 124 patients gathered over 35 years, with clinical features associated with congenital muscular dystrophies. In 41 out of 122 probands (33.6%), the genetic diagnosis was achieved using muscle biopsy and histological examination, immunohistochemical analysis, candidate gene sequencing, and chromosomal microarray (CMA). NGS approaches were performed in 33 out of 124 patients, and 12/22 patients were finally diagnosed with WES. In addition, DNA from the parents was included in 19 cases, 10/11 patients were diagnosed with one of two neuromuscular gene panels (45 and 345 genes, respectively), and WGS was performed in three probands in which diagnosis was not obtained by using WES. Using the WES-based genetic analysis approach, the diagnostic yield was 54%, which was lower compared to the high value of 91% obtained using gene panels. The exceptionally high result for gene panels may be explained by the limited but widely analyzed and well clinically characterized number of cases included in the diagnostic panel [30].

Schofield et al*.* compared the diagnostic yield of traditional gene sequencing (46%) with a genetic panel for NMD, and the diagnostic yield increased up to 75% for genetic panels and up to 79% for WES, assuming that patients diagnosed via candidate gene sequencing would also be diagnosed using CMA plus NMD panel or WES. The study also included financial analysis, which showed the NMD panel to be the most cost-effective approach compared with the traditional NMD diagnostic approaches [28].

Focusing exclusively on the specific type of CMD, Lee et al. studied 15 patients with nemaline myopathy diagnosed previously by pathology, with patients showing typical, intermediate, and mild forms of the disease. A definite genetic diagnosis was confirmed by WES in seven patients, yielding a diagnostic performance of 47%. Pathogenic variants were found in the *NEB* gene in five patients, the *TPM3* gene in one patient, and the *ACTA1* gene in one patient [31].

### Diagnostic yield of exome in muscular dystrophies

Yiş et al*.* studied a total of 56 patients with childhood-onset limb-girdle dystrophy, of which seven patients were evaluated by WES. These seven patients were successfully confirmed for genetic diagnosis after nonspecific muscle biopsy findings, confirming causative variants in one case in *POMT1*, one case in *LMNA*, one patient in *SGCG*, two patients in *SGCB*, and two in *CAPN3* gene [32].

Zamani et al. performed WES in 40 patients clinically suspected of Duchenne muscular dystrophy with negative MLPA analysis, reporting a diagnostic yield of 90% (36/40) patients) and detecting nonsense variants in 63.8%. Unfortunately, four patients remained without confirmatory genetic diagnosis [33]. Luce et al. also studied patients with suspected Duchenne muscular dystrophy without a definitive diagnosis; WES confirmed pathogenic variants in 36 out of 38 cases. DMD was confirmed in 32 boys and was discarded in four patients with absence/deficiency of dystrophin in muscle biopsy, those diagnosed with LGMD causative genes, including the *FKRP* gene in two patients, the *SGCG* gene in one patient, and the *SGCA* gene in one patient. Therefore, the WES diagnostic yield achieved a value as high as 94%. In addition, there is a possibility that patients with muscle biopsy showing abnormalities for dystrophin could have mutations in genes other than the DMD gene [34].

A study of NMD patients with mainly muscular affection was carried out by Božović et al. WES test was performed in 22 pediatric patients evaluated for a variety of muscular diseases including DMD, CM, LGMD, and unspecified myopathy, reaching a notably high diagnostic yield of 64% [35].

### Diagnostic yield of exome in hereditary neuropathies

Walsh et al*.* studied 50 patients with hereditary neuropathies, of whom 23 were pediatric patients. The diagnostic yield of WES in pediatric patients was 47% (7/23). WES was a valid test to diagnose complex phenotypes in a patient with severe early-onset neuropathy, thyroid agenesis, and polydactyly that had a missense variant in the *PMP22* gene consistent with Dejerine-Sottas disease, and another patient with sensory axonal neuropathy, global developmental delay, optic atrophy, and cerebellar ataxia was identified with de novo variant in the *KIF1A* gene [36].

### Diagnostic yield of exome in the prenatal and neonatal onset of NMDs

Disease onset of severe forms of NMD can occur very early in life, manifesting even at the prenatal age. In this regard, the study of Todd et al. included 45 probands from 38 family groups presenting with prenatal and neonatal NMD onset. Consanguinity was reported in 10 pedigrees. The diagnostic yield of WES performed in 23 probands reached 56%. Four novel genes related to neuromuscular diseases were found, consisting of *GPR126*, *KLHL40*, *KLHL41,* and *SPEG* genes. Even more, the study also reported de novo mutations in *CHRND, KLHL40, NEB,* and *RYR1* genes [37].

In Heude et al.’s study, the approach consisted of performing genetic tests on 19 neonates with suspected NMD-related respiratory distress. Five patients (from four families) underwent WES. The diagnostic yield was 20%, obtaining a definite and confirmatory result in one of the five WES patients, a rather low efficiency compared to the data presented [38].

## Discussion

Our review is focused on exome sequencing analysis for NMD diagnosis while nonetheless highlighting the importance of a comprehensive approach for the diagnosis of NMDs. As mentioned, most studies included in this narrative review begin with an exhaustive examination of the patient, family history, laboratory reports, electrophysiological studies, and muscle biopsy when required to achieve deep phenotyping of patients. Once patient anamnesis and phenotype have been fully evaluated, the diagnostic journey requires appropriate genetic testing, including MLPA, single-gene sequencing analysis, targeted clinical exome, NMD gene exome-based panels, WES or WGS, and RNA sequencing.

The diagnostic yield of exome sequencing performed in large groups of non-related patients with a heterogeneous NMD diagnosis has improved in the last few years, yet it remains below 50% [22–24], while in small groups, it can perform slightly better [26]. Consanguinity is reported in population groups studied for NMD [4], and given that the estimated rate of couples related as second cousins or closer is 10.4% worldwide, consanguinity could be a relevant feature in study populations with suspected NMD [39]. In this regard, Fattahi et al. found the highest diagnostic yield of 73% in studies with different types of NMD [25]. This study, in particular, confirmed a high proportion of cases with muscular dystrophies including DMD and LGMD, in which WES gave the highest yield. The finding shared by the five studies with different clinical NMD was the identification of new pathogenic variants and diagnosis of complex and non-neuromuscular diseases.

Studies describing WES diagnostic yield in congenital muscular dystrophies and congenital myopathies [28–30] reported results higher than 50%. However, Masri et al*.* found a diagnostic yield of 43% despite the feature of consanguinity [39]. Tsang et al. found a diagnostic yield of 17% for the subset of congenital myopathies and 45% for the subset of congenital muscular dystrophies [22]. In this line, in the study performed by Lee in only one type of CMD (nemaline myopathy), the diagnostic yield remained below 50% [31], probably as a consequence of the genetic heterogeneity existing in nemaline myopathy [40]. In the appropriate setting, a muscle biopsy study helps to characterize the clinical picture of patients with NMDs, particularly CMD, CM, and DMD, but is nonetheless invasive and painful and could eventually be avoided if WES analysis results in a confirmatory diagnostic for NMDs.

Exome sequencing is not usually included as part of the patient diagnostic journey in Duchenne muscular dystrophy (DMD) because most individuals with this disease (70%) have a single-exon or multi-exon deletion or duplication in the *DMD* gene [41]. Single nucleotide variants distributed in all exons of the *DMD* gene are disease causative in approximately 10% of patients with DMD [42]. Therefore, the first approach is based on dystrophin gene deletion and duplication testing by MLPA or CGH array. When the result is negative, whole sequencing of the *DMD* gene and RNA sequencing from muscle tissue can detect rare deep intronic variants or structural variants affecting the *DMD* gene [41]. As expected, the diagnostic yield of WES reported in the DMD phenotype is above 90%, although never reaching 100%. In this regard, clinicians should evaluate whether WES-based genetic analysis will provide relevant information about other pathogenic variants of the *DMD* gene or contribute to diagnosing other NMDs. In cases where WES is performed in patients affected by DMD, this approach enables the confirmation of specific variants and the identification of patients who consequently benefit from specific treatment.

In the Walsh study of hereditary peripheral neuropathies, WES was able to confirm the diagnosis of patients with complex phenotypes [36]. Hereditary peripheral neuropathies are among the most frequent genetic neuromuscular disorders, whose genotypic and phenotypic variability constitute a diagnostic challenge involving more than 100 causative genes [43].

Precise diagnosis is part of the integral care of pediatric patients affected by rare genetic diseases. Scientific literature shows the diagnostic utility of exome analysis at various times during the patient’s diagnostic journey, both at baseline studies and during patient follow-up when possible causal genes have been ruled out or even at the end of a difficult diagnostic odyssey [44]. With early and accurate diagnosis of a patient suffering from an NMD, physicians can provide an exact prognosis and guidance on clinical treatment, give family counseling, and can even optimize patient follow-up to detect NMD-related complications. Positive or negative results derived from WES regarding the genetic cause of a disease will impact the clinical management of NMD patients [45].

Sometimes, patients may have a dual molecular diagnosis. For example, this situation occurred in two patients analyzed in the Herman et al. study. One patient had pathogenic variants in *MAGEL2* and *CHRND*, which are diagnostic for Schaaf-Yang syndrome and congenital myasthenic syndrome, respectively. Another patient showed composed heterozygosity for both *RYR1* and *TTN*, which supports diagnoses of two NMD, central core disease and limb-girdle dystrophy, respectively [23]. Hypotonia and muscle weakness on the physical examination do not always lead to a clinical diagnosis for a specific NMD. In the study of Kuperberg et al., there were two cases characterized by muscle weakness, but the final diagnosis identified these two cases as non-neuromuscular diseases after WES. In the first case, the patient was diagnosed with Schwachman-Diamond syndrome, and in the second patient, a mutation in the *TGBF1* gene which causes Camerutti-Engelman syndrome was found, an autosomal disorder characterized by bone dysplasia and pain [26]; also, in the Tsang’s study, a patient was detected with Camerutti-Engelman syndrome [22].

**Fig. 2:**
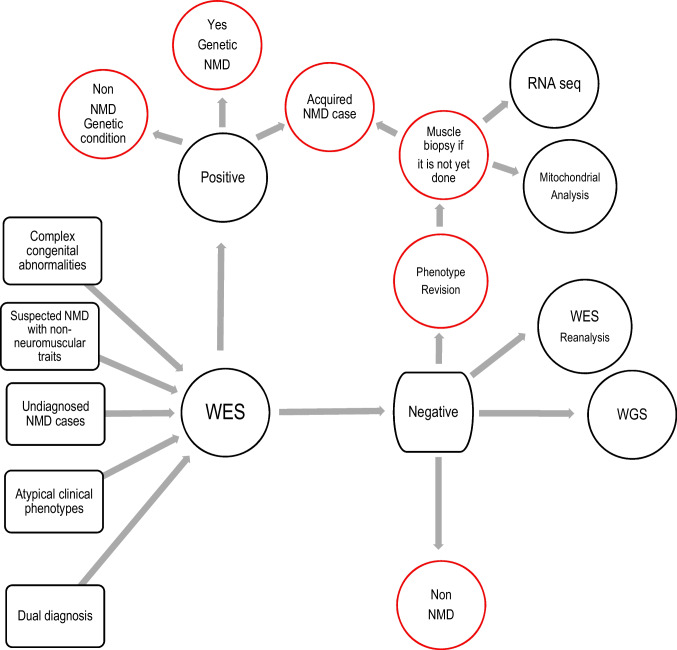
Flowchart for WES in NMD and downstream approaches when WES does not provide a successful diagnosis. A negative diagnosis for WES should be followed by other approaches such as WES reanalysis, RNA-seq, WGS, and mitochondrial analysis. Positive results are sometimes reached after complementary studies that include the Sanger sequence of the gene of interest. Note that sometimes the exome will be positive for non-NMD conditions or can be positive, but the patient’s disease is the result of an acquired NMD or with a negative result because the patient does not have NMD

WES is a useful diagnostic tool for genetic NMDs when a diagnosis has not been obtained via other genetic analysis tests and as a first-tier test in cases of atypical clinical phenotypes, suspected dual diagnosis, complex congenital abnormalities, non-neuromuscular disorders, or undiagnosed cases. After a negative exome result, deep phenotyping may allow to identify those patients who do not have a genetic neuromuscular disease or those patients who even having a pathogenic or causal variant, present a neuromuscular disease that is explained by a toxic, inflammatory, or autoimmune mechanism (Fig. 2). The developing field of gene therapy opens up an opportunity to provide patients with accurate diagnoses to receive treatment that can alter the natural history of not only single-gene approach diseases such as DMD and SMA5q- but also heterogeneous ones such as ALS and also has potential for treatment of many other NMDs in the future.

Finally, the current challenge is to narrow the gap of the WES test as regards the diagnosis rate in a cost-effective way by careful planning and selecting each analysis since any positive or negative result of WES will impact the patient and family. In line with the rapidly advancing knowledge of the pathogenesis of disease gene variants, upgrading variants of uncertain significance and clinical updates, and performing WES reanalysis with the appropriate timing and other diagnostic tools such as whole-genome sequencing (WGS), RNA-seq, and protein analysis expression will all serve to increase diagnostic yield in NMD.

This study has some limitations; first, we are aware about the heterogeneity of diagnosis and studies included in this review, which come from the different techniques, commercial kits, mean deep, and coverage of exome analysis and differ among the studies included in this review. The second limitation to consider is that some studies included in this review were focused on both children and adult patients affected by neuromuscular diseases.

## Conclusions

Despite valuable research contributions in reporting NMD-related genes, the yield of WES for diagnosis of NMD patients currently remains around 50%, except in certain settings such as single-gene diseases, known disease-causative genes, and well-studied patient cohorts. In this regard, WES and gene panels have similar diagnostic yields in patients with NMD, particularly in CMD, partly because genetic panels are exome-based and WES analyzes only coding genome sequences. Taken together with the possibility of unsuccessful results, this indicates a need for complementary analysis, combining strategies or performing WES reanalysis to obtain an accurate final diagnosis. WES misses structural rearrangements, copy number variants, and repetition expansions, which limits the information that can be obtained from this strategy. The existence of gene variants not yet associated with disease, along with the inadequate coverage of intronic and regulatory regions by WES, indicates room for improvement and points to future directions in research.

## Funding 

This work was funded by CIBERER Cooperative and complementary intramural actions (ACCI) ENoD 2019 and EpiENoD 2022 and AES2019 (ISCIII) project PI19/00994.

## Data Availability

Not applicable.
